# Activation PDGFR-α/AKT Mediated Signaling Pathways in Oral Squamous Cell Carcinoma by Mesenchymal Stem/Stromal Cells Promotes Anti-apoptosis and Decreased Sensitivity to Cisplatin

**DOI:** 10.3389/fonc.2020.00552

**Published:** 2020-04-28

**Authors:** Jia Wang, Ruwen Cui, Cecila G. Clement, Ranjana Nawgiri, Don W. Powell, Irina V. Pinchuk, Tammara L. Watts

**Affiliations:** ^1^Department of Otolaryngology, University of Texas Medical Branch, Galveston, TX, United States; ^2^Department of Pathology, University of Texas Medical Branch, Galveston, TX, United States; ^3^Department of Internal Medicine, Division of Gastroenterology, University of Texas Medical Branch, Galveston, TX, United States; ^4^Department of Microbiology and Immunology, University of Texas Medical Branch, Galveston, TX, United States

**Keywords:** PDGFR-α, AKT, oral cancer, crenolanib, apoptosis, mesenchymal stem cells, cisplatin

## Abstract

Desmoplasia, a hallmark of a head and neck cancer, has both biologic and physiologic effects on cancer progression and chemotherapeutic response. Mesenchymal stem/stromal cells (MSCs), also known as mesenchymal stromal progenitor cells, have been shown to play a role in cancer progression, alter apoptotic responses, and confer resistance to chemotherapy in various carcinomas. The pathophysiology of MSCs with respect to tumorigenesis is widely reported in other cancers and is sparsely reported in oral squamous cell carcinomas (OSCCs). We previously reported paracrine mediated PDGF-AA/PDGFR-α signaling to underlie MSCs chemotaxis in OSCC. Given the poor clinical response to primary chemotherapy, we hypothesized that MSCs may alter cancer cell sensitivity to cisplatin through activation of PDGFR-α mediated signaling pathways. Co-culture of MSCs with human derived OSCC cell lines, JHU-012 and −019, resulted in a significant increase in the production of PDGF-AA and MCP-1 compared to cancer cells grown alone (*p* < 0.005) and was accompanied by an increase in the phosphorylation state of PDGFR-α (*p* < 0.02) and downstream target AKT at S473 (*p* < 0.025) and T308 (*p* < 0.02). JHU-012 and −019 cancer cells grown in co-culture were significantly less apoptotic (*p* < 0.001), expressed significantly higher levels of Bcl-2 (*p* < 0.04) with a concomitant significant decrease in bid expression (*p* < 0.001) compared to cancer cells grown alone. There was a significant increase in the cisplatin dose response curve in cancer cell clones derived from JHU-012 and 019 cancer cells grown in co-culture with MSCs compared to clones derived from cancer cells grown alone (*p* < 0.001). Moreover clones derived from JHU-012 cells grown in co-culture with MSCs were significantly more susceptible to cisplatin following pretreatment with, crenolanib, a PDGFR inhibitor, compared to cancer cells grown alone or in co-culture with MSCs (*p* < 0.0001). These findings suggest that crosstalk between cancer cells and MSCs is mediated, at least in part, by activation of autocrine PDGF-AA/PDGFR-α loop driving AKT-mediated signaling pathways, resulting in reduced cancer cell sensitivity to cisplatin through alterations in apoptosis.

## Introduction

Mesenchymal stem/stromal cells (MSCs) have been recognized to play important roles in the pathogenesis and progression of several solid cancers, including breast, prostate, non-small cell lung cancer, and others (1–5). The robust desmoplastic reaction characteristic of pancreatic cancer, and recent reports that MSCs in the pancreatic cancer stroma have tumor promoting properties, highlights the importance of MSCs in this context (6). Akin to pancreatic cancer, head and neck squamous cell carcinomas (HNSCC), of which the majority arise from the oral cavity and oropharyngx, are characterized as having an extensive desmoplastic response, suggesting that the tumor stroma may play an important role in the pathophysiology of this cancer as well (7). Although the source of mesenchymal cells in the tumor stroma are numerous, several lines of evidence support MSCs as an important source of desmoplastic fibrocytes in the TME (8, 9).

In one of the first reports to identify and detail a mechanism for MSC homing to the TME of HNSCC, we reported that MSCs home to the TME of oral cavity and oropharyngeal squamous cell carcinoma through the chemotactic action of PDGF-AA mediated through PDGFR-α (10). Our work expanded on initial reports by Liotta et al. who isolated CD90^+^ tumor associated MSCs and showed that they promote tumor suppression by modulating T-cell responsiveness (11). Moreover, Liu et al. reported bone marrow derived MSCs promote oral cavity cancer progression via periostin-mediated activation of PI3K/AKT signaling pathways resulting in reduced cancer cell apoptosis (12).

Activation of AKT-mediated signaling pathways has been shown to play a pivotal role in cancer progression by promoting cancer cell survival by several mechanisms including altering apoptotic response (13). Activation of AKT allows Bcl-2 to outcompete Bax, resulting in an anti-apoptotic response and upregulation of Bcl-2 has been shown to be an important mechanism of chemo-resistance in cancer cells (14, 15). Bcl-2 expression is routinely reported in HNSCC surgical pathology specimens, has been shown to be overexpressed in patients with HNSCC (16), and overexpression in early HNSCC correlates with a significant decrease in 5-year disease free recurrence and overall survival for patients treated with primary radiation (17). Expression of Bcl-2 and activation of AKT are known pathways affecting cisplatin mediated cytotoxicity (18). Park et al. reported overcoming cisplatin resistance by downregulating Bcl-2 in HNSCC by modulating tristetraprolin (TTP) expression (19), the mechanisms underlying linking Bcl-2 overexpression and therefore reduced sensitivity to cisplatin in HNSCC have not been reported.

Resistance to chemotherapy is a multifactorial process. Growing evidence suggests that cancer stem cells (CSCs), cancer associated fibroblasts (CAFs), and MSCs (which differentiate into CAFs in the TME) play an important role in the development of *de novo* chemo-resistance (4, 20–24). CAFs have been shown to promote decreased sensitivity to gemcitabine in pancreatic cancer (25). Moreover, in non-small cell lung cancer, activation of AKT/Sox2 pathway by CAFs induced cancer cell resistance to chemotherapy (26). Given our recent findings that MSCs home to the TME in oral cavity and oropharyngeal cancer, collectively here referred to as oral squamous cell carcinoma (OSCC) and the recent reports of the role of MSCs in the context of chemotherapy resistance to platinum based agents, we sought to understand if crosstalk between MSCs and oral squamous cell carcinoma cells is mediated by PDGFRα/AKT signaling may be implicated in cisplatin resistance through changes in cancer cell apoptosis.

## Methods

### Cell Culture

Head and neck cancer cell lines JHU-012, JHU-019 (derived from human oropharyngeal tumors) and OKF-TERT1 human immortalized non-neoplastic oral keratinocyte cells (OKT) were generously provided by Dr. Vicente Resto (Galveston, TX). Cells were maintained in RPMI 1640 medium containing glutamine supplemented with 10% fetal bovine serum at 37°C in 5% CO_2_. Primary bone marrow-derived human mesenchymal stem cells (MSCs) were obtained from ATCC (Manassas, VA) and maintained according to the manufacturer's recommendations. MSCs were used between passages 2–5 and defined as early passage. The human OPSCC cell lines used in these studies have been extensively characterized both *in vitro* and *in vivo* (27, 28). For co-culture conditions, MSCs and HNSCC cell lines JHU-012, JHU-019, and negative OKT controls were grown in a 1:1 and supplemented in 1:1 ratio of appropriate culture media for 6 days.

### Cell Viability, Apoptosis and Cell Proliferation

Cell viability was measured using the XTT cell viability kit (Cell Signaling Tech., 9095) in 96 well plates at 2 x 10^3^ cells per well following manufacturer's protocol. Apoptosis was measured by flow cytometry analysis with the ANXA5/PE/7-AAD Apoptosis Detection Kit (BD Biosciences) at 1 x 10^6^ cells per falcon tube. Prior to apoptosis detection, cells were stained with APC-anti-human CD326 (EpCAM) Clone:CO17-1A (Biolegend) to detect epithelial cells and PE/Cy7 anti-human CD90 (Thy1) Clone:5E10 to detect human MSCs. Cell acquisition was performed on BD LSRFortessa™ cell analyzer (BD Biosciences) at UTMB Flow Cytometry Core Facility in the Department of Microbiology and Immunology. Cellular proliferation was measured by BrdU - ELISA (colorimetric) assay (Abcam) in a 96 well plate at 2 x 10^3^ cells per well with 4 h BrdU incubation period.

### Transwell Studies

To determine if MSCs mediated alterations in cancer cell apoptosis required direct contact transwell co-culture assays were conducted as previously described (2). Briefly, 2.5 x 10^5^ MSCs were seeded on the filter in the upper chamber of the transwell filter with a pore size of 8.0 μm in a final volume of 250 μl and 2.5 x 10^5^ JHU-012 or OKT cells were seeded on the bottom chamber. Following 6 days of co-culture, apoptosis of the epithelial cells was measured as described above.

### Clonogenic Assay

Clonogenic assay conditions were established as previously described (29). HNSCC cell line JHU-012 and −019 alone or in co-culture with MSCs, as described above, were seeded in 6-well plates at predetermined densities. Cells were allowed to settle and adhere for 24-h, prior to treatment with cisplatin. Cells were treated with varying doses of cisplatin ranging from 0 to 4 μM for 24 h. After 24-h treatment with cisplatin, the media was exchanged and with basal media and cultured 5 additional days. Cells were fixed with 3% crystal violet/10% formalin, imaged and counted using ImageJ.

### Western Immunoblotting

OSCC cells lines JHU-012 and JHU-019 were cultured alone or co-cultured with MSCs at 1:1 ratio in media containing 50% of RPMI 1640 complete medium (Thermo Fisher Scientific, MA) and 50% of MSC Complete Medium (ATCC, VA) for 6 days. Cells were lysed in 1 x RIPA buffer (Cell Signaling Technology, MA) with protease inhibitor cocktail (Sigma-Aldrich, MA). Protein concentrations were measured using BCA protein assay (Thermo Fisher Scientific). Cell lysates (10 μg protein) were separated by NuPAGE 4–12% Bis-Tris protein gels (Thermo Fisher Scientific) and transferred onto PVDF membranes (Bio-Rad, CA). After blocking with 5% (w/v) nonfat dry milk in TBS-T, the membranes were incubated with primary antibodies for anti-PDGFR-α (phosphoY754; abcam #ab5460) p-AKT (Thr308) (#13038, Cell Signaling Technology), p-AKT (Ser473) (#4060, Cell Signaling Technology), total AKT (#4691, Cell Signaling Technology), and β-actin (#A1978, Sigma-Aldrich). Antibody dilution ranged from 1:500 to 1:1000. After three washes with TBS-T, the membranes were incubated with the appropriate secondary antibodies. Reactive protein bands were visualized using Alpha Innotech Fluorchem FC2 imaging system (Cell Bioscience, CA). The intensity of each protein band was quantified by Image J software (NIH).

### Quantification of Chemokines

Cytokine production was measured in the supernatants of the conditioned media from OSCC cell lines JHU-012, and −019 Human XL cytokine discovery premixed kit (R and D Systems, FCSTM18, Minneapolis, MN) according to the manufacturer's instructions.

### Statistical Analysis

Each experiment was with *n* ≥ 3 in triplicate, unless otherwise noted. All data were expressed as mean ± standard error of mean (SEM). Statistical analyses were generated using GraphPad Prism (GraphPad Software 7.0) or Excel 2013 (Microsoft Excel Spreadsheet Software 2013). For the comparison between two or more experimental groups, statistical significance was assessed via Student's *t*-test or two-way ANOVA with *p*-value < 0.05 being considered statistically significant. Dose response curves were generated using GraphPad Prism set with the following parameters: [drug] vs response variable slope (four parameters). Extra sum of squares F test was used to reject the null hypothesis with *p* < 0.05 being considered statistically significant. Each dose response curve was done in triplicate with *n* = 3.

## Results

### MSCs Activate a PDGF-AA/PDGFR-α Autocrine Loop in Oral Squamous Cell Carcinoma (OSCC)

**Figure 1:**
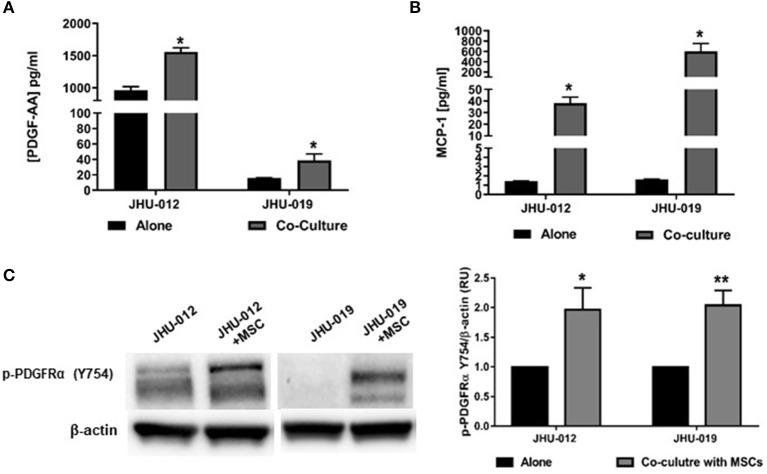
MSCs induce increased production of PDGF-AA and MCP-1 and Autocrine Activation of PDGFR-α in OSCC. JHU-012, and −019 were grown alone or in 1:1 co-culture with MSCs for 6 days and production of PDGF-AA and MCP-1 measured using a multiplex assay approach and activation of PDGFR-α determined by Western immunoblotting. There was a significant increase in the production of PDGFAA **(A)** and MCP-1 **(B)** in the conditioned media from cancer cells grown in co-cultured compared to cancer cells grown alone (*n* = 3, *p* < 0.005). **(C)** Following co-culture with MSCs, there was a significant increase in expression of p-PDGFR-α (Y754) in JHU-012 and −019 co-cultures (*n* = 3; *p < 0.02; **p < 0.006).

To investigate whether MSCs induce changes in PDGF-AA/PDFGR-α expression with activation of downstream signaling targets in OSCC cells, JHU-012 and −019 were grown in 1:1 co-culture with early passage human MSCs for 6 days. The conditioned media from OSCC cells grown alone or in co-culture with MSCs was screened using a multiplex approach for cytokines previously shown to be upregulated in human cancers. Of the 45 cytokines assayed, only PDGF-AA (Figure 1A) and MCP-1 (Figure 1B) were found to be significantly increased the conditioned media from OSCC cells grown in co-culture compared to OSCC cells grown alone (*p* < 0.005). There was no appreciable secretion of PDGF-AA, MCP-1 in MSCs grown alone nor appreciable secretion of PDGF-AB/BB in either MSCs, OSCC cells grown alone, or in co-culture with MSCs (data not shown). To investigate whether increased production of PDGF-AA resulted in activation of PDGFR-α, the expression of phosphor-PDGFR-α was determined by Western immunoblotting. There was a significant increase in phosphorylation of PDGFR-α in JHU-012 (*p* < 0.02) and −019 (*p* < 0.006) co-cultured with MSCs compared to cancer cells grown alone (Figure 1C). We did not detect appreciable expression of total PDGFR-α by Western immunoblotting (Supplemental Figure 1).

**Figure 2:**
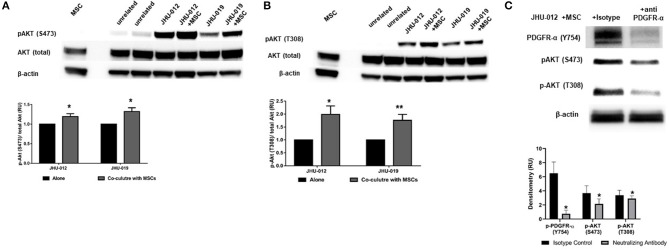
MSCs induce activation (phosphorylation) of PDGFR-α with downstream activation of AKT in OSCC. **(A)** Downstream activation of p-AKT levels were observed at serine 473 (S473) in co-cultures from JHU-012, and −019 compared to cancer cells grown alone. (*n* = 3; *p* < 0.025). **(B)** A more robust increase in p-AKT levels were also observed at threonine 308 (T308), corresponding to the catalytic site for AKT in JHU-012 and −019 grown in co-culture with MSCs (*n* = 3; *p < 0.02; **p < 0.007). **(C)** Inhibition of PDGFR-α results in decreased expression of activated AKT. JHU-012 were grown alone or in 1:1 co-culture with MSCs for 6 days in the presence of PDGFR- α neutralizing antibodies or isotype control and expression of p-PDGFR-α (Y754), pAKT (S473) and p-AKT (T308) measured by Western immunoblotting. There was a significant decrease in p-PDGFR-α, p-AKT (S473), and pAKT (T308) expression following inhibition of PDGFR-α neutralizing antibodies compared to isotype control (*n* = 3; *p* < 0.03).

Activation of PDGFRs have previously been shown to be an important regulator of Akt activation. Therefore to determine if activation of MSCs induced activation of PDGFR-α in OSCC cells affects AKT expression, p-AKT was assessed by Western immunoblotting. Downstream p-AKT expression was found to be significantly increased in OSCC co-cultures of these same cancer cells lines at S473 (Figure 2A; *p* < 0.025) and T308 (Figure 2B; *p* < 0.02); suggesting the presence of MSCs in co-culture with JHU-012 and −019 results in activation of PDGFR-α/AKT mediated signaling pathways. To further confirm the specificity of activation of PDGFR-α in mediating changes in AKT expression, JHU-012 cells were grown alone and in co-culture with MSCs in the presence of PDGFR-α neutralizing antibody and isotype control (Figure 2C). Following treatment with PDGFR-α neutralizing antibodies, there was a significant decrease in p-PDGFR-α expression in JHU-012/MSC co-cultures (*p* < 0.03) with a concomitant significant decrease in p-AKT at S473 (*p* < 0.03) and at T308 (*p* < 0.03) compared to isotype control (Figure 2C). Further, we have preliminary evidence for high expression of levels of PDGFR-α on both tumor epithelial cells and tumor stromal cells in patients with advanced OSCC, further suggesting activation of autocrine PDGF-AA/PDGFR-α loop between MSCs and OSCC (30).

### MSCs Mediate Increased Expression of Bcl-2, Decreased Expression Bid and Reduced Apoptosis in OSCC

**Figure 3:**
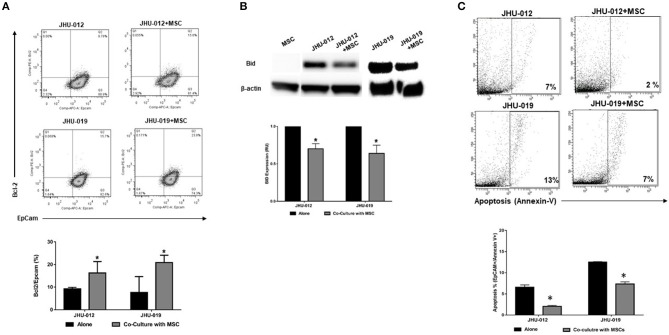
MSCs mediate increased expression of Bcl-2, decreased expression of Bid, and decreased apoptosis in OSCC. JHU-012, and −019 were grown in a 1:1 co-culture with MSCs for 6 days. Bcl-2, Bid and apoptosis expression measured. EpCAM was used to identify cells of epithelial lineage and CD90 used to identify mesenchymal cells. **(A)** There was a significant increase in the expression of bcl-2/EpCAM^+^ cells in co-cultures derived from JHU-012 and −019 compared to cancer cells grown alone (*n* = 3; *p* < 0.04). **(B)** There was a significant reduction in bid expression across in JHU-012 and −019 co-cultured with MSCs compared to cancer cells grown alone (*n* = 3; *p* < 0.001). **(C)** There was a significant reduction in the number EpCAM^+^/annexin V^+^ cancer cells undergoing apoptosis in the co-cultures derived from JHU-012 and JHU-019 compared to cancer cells grown alone (*p < 0.001, *n* ≥ 3).

To determine if activation of AKT by MSCs results in alterations in apoptotic responses in OSCC cells, bcl-2 expression was measured by flow cytometry. Co-cultures were sorted based on expression of the epithelial lineage marker EpCAM and the MSC lineage marker CD90. Following 6-days of co-culture, there was a significant increase in the expression of bcl-2 in EpCAM^+^ JHU-012 and −019 cells grown in co-culture with MSCs (Figure 3A; *p* < 0.04). In addition, expression of the pro-apoptotic protein Bid was significantly reduced in JHU-012 and −019 cells co-cultured with MSCs compared to cancer cells grown alone (Figures 3B,C; *p* < 0.001). Further, there was a significant reduction in apoptosis in EpCAM^+^/annexin V^+^ JHU-012 and −019 co-cultures (Figure 3C; *p* < 0.001). These data suggest that MSCs reduce apoptotic response in OSCC cells *in vitro* by modulating expression of the Akt target bcl-2 in JHU-012 and -019.

### MSCs Mediate Decreased Cytotoxicity to Cisplatin in OSCC

**Figure 4:**
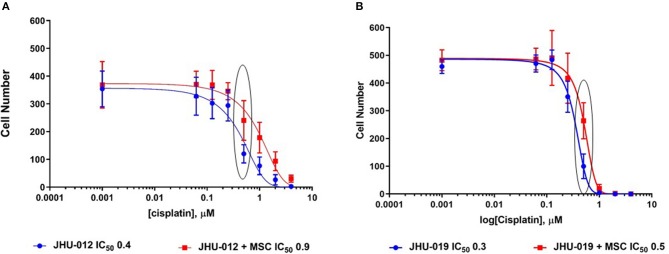
MSCs mediate decreased cytotoxicity to cisplatin in OSCC. Using a clonogenic assay approach, cisplatin dose response curves were generated for clones derived from JHU-012 and −019 cells grown alone and in 1:1 co-culture with MSCs. There was a significant increase in the cisplatin dose response curve and IC_50_ in clones derived from co-culture with MSCs for JHU-012 **(A)** and −019 (**B**; *n* > 3; *p* < 0.001).

**Figure 5:**
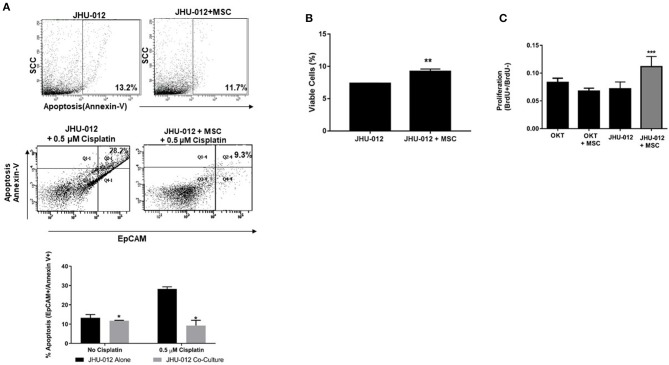
MSCs mediate decreased apoptosis with a concomitant increase cell viability and proliferation following 0.5 μM treatment with cisplatin in OSCC. JHU-012 cells grown alone or in 1:1 co-culture with MSCs were treated with 0.5 μM cisplatin and apoptosis, viability, and proliferation measured. **(A)** 1:1 co-culture of MSCs with JHU-012 resulted in a significant decrease in EpCAM+/annexin V+ cancer cells undergoing apoptosis compared to JHU-012 cells grown alone (*p < 0.0001, *n* = 3). **(B)** JHU-012/MSC co-cultures displayed enhanced viability compared to cancer cells alone (**p < 0.001, *n* = 3) and **(C)** cancer cell proliferation was significantly increased following cisplatin treatment in co-culture conditions compared to cancer cells grown alone and OKT negative controls grown alone or in co-culture with MSCs (***p < 0.026, *n* = 3).

Cisplatin-mediated cytotoxicity occurs through crosslinking of DNA, resulting in loss of DNA repair and the initiation of apoptosis (31). Therefore, we hypothesized that MSC-mediated OSCC cell response to apoptosis may alter cancer cell cytotoxicity to cisplatin. Using a clonogenic assay approach, single clones were isolated from JHU-012 and −019 grown alone or derived from cancer cells grown in 1:1 co-culture with MSCs. Cells were treated with varying doses of cisplatin for 24-h, as previously described (32). There was a significant increase in the dose response curves and IC_50_ from clones derived from JHU-012 (Figure 4A; *p* < 0.001) and −019 (Figure 4B; *p* < 0.001) grown in co-culture with MSCs, signifying MSCs confer decreased cancer cells sensitivity to cisplatin. The IC_50_, a log-based value, for cisplatin in clones derived from JHU-012 grown in co-culture with MSCs was >2 times higher (0.9 vs. 0.4) than the IC_50_ in clones derived from JHU-012 grown alone (Figure 4A). While slightly lower at 1.6 times higher (0.5 vs 0.3), a similar significant trend in the IC_50_ for cisplatin was observed in clones derived from JHU-019 grown in co-culture with MSCs compared to JHU-019 grown alone (Figure 4B). Representative crystal violet staining patterns for cisplatin colongenic assays are depicted for JHU-012 and −019 are provided in Supplemental Figures 2A,B, respectively. In addition, following treatment with 0.5 μM cisplatin, JHU-012 cells grown in co-culture with MSCs were significantly less apoptotic with increased cell viability and proliferation compared to cancer cells grown alone (Figure 5, *p* < 0.0001). These data support that crosstalk between MSCs OSCC cells as a mechanism mediating altered cisplatin response in oral cancer as seen in other desmoplastic rich cancers (33).

### Inhibition of PDGFR-α With Crenolanib Decreases Expression of Activated PDGFR-α and Improves Responsiveness to Cisplatin in OSCC

**Figure 6:**
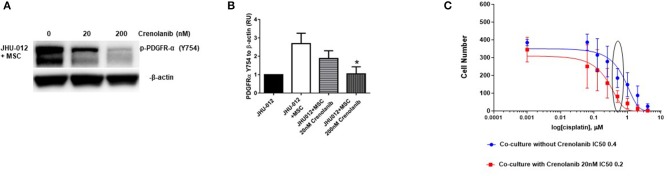
Inhibition of PDGFR-α with crenolanib decreases expression of activated PDGFR-α and improves responsiveness to cisplatin in OSCC. **(A,B)** JHU-012 were grown in 1:1 co-culture with MSCs in the presence and absence of the receptor tyrosine kinase inhibitor, crenolanib, for 6 days and PDGFR-α expression measured by Western immunoblotting. PDGFR-α expression was decreased following 20 nM treatment and 200 nM treatment with crenolanib. Expression levels were quantified by densitometry which revealed a significant decrease in PDGFR-α expression following treatment with 200 nM crenolanib (*n* = 3; *p < 0.04). Using a clonogenic assay, cisplatin dose response curves were generated from clones grown from JHU-012 **(C)** grown in 1:1 co-culture with MSCs with and without 20 nM crenolanib. There was a significant shift in the cisplatin dose response curve and the IC_50_ was reduced by half in JHU-012 (*p* < 0.0001) cancer cell clones treated with 20 nM crenolanib compared to untreated cancer cell clones (*n* ≤ 3).

PDGFR-α expression was attenuated following treatment with 20 nM crenolanib reaching statistical significance at 200 nM crenolanib (Figures 6A,B; *p* < 0.04). We did not detect appreciable expression of total PDGFR-α by Western immunoblotting (Supplemental Figure 3). In clinical trials the dosing of crenolanib ranges between ~100 and 600 nM. Prior studies suggest 20 nM crenolanib is sufficient to inhibit FLT3 (type-III tyrosine kinase inhibitor) in acute myeloid leukemia (34). Therefore, we selected 20 nM crenolanib as a more physiologic relevant concentration to determine if pretreated with this monoclonal antibody would attenuate the increase in IC_50_ in JHU-012 cells grown in co-culture with MSCs. Cancer cell clones derived from JHU-012 co-cultures were treated with and without 20 nM crenolanib for 6 days and the dose response to cisplatin measured, as previously reported. There was a significant downward shift in the cisplatin dose response curve and a reduction in the IC_50_ by half (0.4 vs. 0.2) in JHU-012 (*p* < 0.0001) clones derived from co-cultures treated with 20 nM crenolanib compared controls (Figure 6C). Representative crystal violet staining patterns for cisplatin colongenic assays are depicted for JHU-012 are provided in Supplemental Figure 4.

## Discussion

We demonstrate in this study a novel mechanism in OSCC cells induced by MSCs resulting in activation of PDGFR-α/AKT mediated signaling pathways resulting in decreased cancer cell apoptosis and reduced cytotoxicity to cisplatin. Moreover, we further show the activation of PDGFR-α mediated signaling pathways and alterations in cisplatin response induced by MSCs in OSCC cells can be overcome by the PDGFR inhibitor, crenolanib. Autocrine PDGFR-α expression has been shown to be upregulated in ovarian cancer and mammary cancer metastasis and expression levels correlated with tumor progression (35, 36). Matei et al. report 39% of ovarian tumors to be PDGFR-α positive and inhibition of the PDGF-PDGFFR axis with imatinab restricted ovarian cancer cell growth *in vitro* (35, 37). In addition, ovarian cancer cells transfected to constitutively express AKT were significantly more resistant to imatinab which could only be partially reversed with high concentrations of imatinab (37).

We recently reported that MSC-mediated chemotaxis in oral cancer cells is dependent upon cancer cell secretion of PDGF-AA acting on PDGFR-α+ MSCs (10). In that study, JHU-012 and −019 we did not detect PDFGR-α expression by Western immunoblotting, leading us to conclude paracrine signaling between OSCC cells and MSCs promotes MSC chemotaxis *in vitro* (10). The physiologic consequence of MSC chemotaxis in this setting was not known. However, in this study using co-culture of MSC with cancer epithelial cells, we conclude that MSCs induce activation of PDGFR-α on OSCC cells resulting in activation of AKT, reduced apoptotic response and decreased sensitivity to cisplatin. Our *in vitro* data is supported by *ex vivo* immunohistochemistry studies which show high expression of PDGFR-α on both tumor cells and TME stromal cells in patients with oral cancers (30), which has not been previously reported in this context. Ongkeko et al. describe high expression of PDGFR in head and neck specimens, however in review of their methods, antibodies staining was noted to be for PDGFR-β and the surrounding stromal cells appear to be PDGFR-β negative (38). This distinction is important as we previously reported inhibition of PDGF-β did not affect MSC chemotaxis by OSCC cells (10). Moreover, TME expression of PDGFR-α has been shown to correlate with a worse prognosis in patients with prostate, breast, ovarian, non-small cell lung cancer and osteosarcoma and we have preliminary data that suggests the TME PDGFR-α may bear prognostic significance in OSCC (4, 35, 39, 40), cancers previous described to have reactive stromal microenvironments implicated in disease progression (41, 42). Activation of PDGFR signaling pathways have been shown to play a significant role in desmoplastic response, which is a hallmark feature of OSCC. Studies are beginning to emerge suggesting bone marrow derived mesenchymal cells may be an important source of circulating stromal cells in TME (3, 9, 41) and autocrine activation of PDGFR-α has been shown to promote epithelial ovarian cancer cell proliferation and thought to play a role in metaplastic transformation of mullerian epithelium (35). Our observations are further supported by Ong et al. who recently reported overexpression of PDGFR-α mRNA was associated with advanced disease in patients with oral cavity squamous cell carcinoma (43, 44).

The clinicopathologic response for patients with advanced OSCC to conventional protocols including surgical excision, chemotherapy, and radiation is poor, suggesting that treatment strategies directed at tumor cells alone are inadequate. Single use biologic agents such as cetuximab have shown poor clinical efficacy, and recently, Braig et al. reported that cetuximab resistance is linked to a single polymorphism in the EGFR (k-allele), which is harbored by >40% of patients with HNSCC (45). Immunotherapy based targeted treatment for oral cancers is in its infancy, with pembrolizumab and nivolumab gaining approval in late 2017 for platinum refractory patients with advanced HNSCC (46).

Immunotherapy and treatments that target non-cancerous cells in the TME, such as MSCs and desmoplastic fibrocytes (i.e., CAFs), may improve clinical outcomes. Crenolanib is a small molecule tyrosine kinase inhibitor with that is effective against PDGFR-α and presently in clinical trials for patients with gastrointestinal stromal tumors (GIST) in which PDGFR*A* activation mutations confer resistance to imatinab (47). Use of receptor tyrosine kinase inhibitors like imatinib have been shown to improve responsiveness to doxorubicin through modulation of AKT signaling in head and neck cancer cells (48), suggesting small molecules like crenolanib may offer therapeutic value in patients with PDGFR-α+ oral cancers.

Activation of PDGR's leads to activation of several downstream events including the PI3K/AKT signaling pathway (49). The AKT pathway is frequently dysregulated in the setting of cancer, and activation of AKT results in an anti-apoptotic phenotype with increased expression of bcl-2 (50). We provide strong evidence that co-culture with MSCs results in increased expression of bcl-2 in oral cancer cells (Figure 2A), and these events appear to be mediated via activation of AKT at T308 and S473 (Figures 1B,C). Vincent et al. report that increased p-AKT at T308 bears prognostic significance in non-small cell lung cancer (51). Phosphorylation of T308 has been found to be essential for catalytic activity and S473 site required for maximal activation (52). In our model, we have found increase p-PDGFR-α and p-AKT at both sites for JHU-012 and -019.

Therefore, we hypothesized that disruption of the PDGF-AA/PDGFR-α signaling between OSCC cells and MSCs with the receptor tyrosine kinase inhibitor crenolanib, may restore cancer cell sensitivity to cisplatin. Crenolanib is presently in active clinical trials for both hematologic and solid tumors and has been shown to be 100-times more selective for PDGFR-α compared to other agents (34, 47, 53, 54). We show here that treatment with crenolanib blocks activation of PDGFR-α (Figure 6A) and pretreated with 20 nM crenolanib significantly reduces the cisplatin dose response OSCCC cells grown in co-culture with MSCs compared to untreated MSC oral cancer cell co-cultures (Figure 6C; *p* < 0.0001). Pretreatment with of MSC oral cancer co-cultures with crenolanib restored cisplatin sensitivity to that of cancer cells grown alone.

Given the widespread use of cisplatin in the management of advanced OSCC, understanding how MSCs mediate resistance to this agent has translational relevance. In addition to these novel findings with respect to MSCs contribution to cisplatin resistance through anti-apoptosis in oral cancers, we also demonstrate this resistance can be overcome by a small molecule PDGFR-α inhibitor crenolanib. The emerging role of MSCs in pathophysiology of cancer, metastasis, and drug resistance has translational relevance and may affect patient outcomes. We have demonstrated here that MSCs contribute to anti-apoptosis and resistance to cisplatin in OSCC *in vitro*. Our findings suggest selective targeting of TME MSCs may provide a viable treatment strategy to combat cisplatin resistance and warrants further mechanistic and translational study in OSCC.

## Data Availability Statement

All datasets generated for this study are included in the article/Supplementary Material.

## Author Contributions

JW and RC: study design, collection and/or assembly of data, and data analysis. CC and RN: blinded pathologic grading of specimens. IP and DP: study design, data analysis and interpretation, manuscript review. TW: conception and study design, collection and assembly of data, data analysis and interpretation, manuscript preparation, and final approval.

## Conflict of Interest

The authors declare that the research was conducted in the absence of any commercial or financial relationships that could be construed as a potential conflict of interest.

## Supplementary Material

Supplemental Figure 1 Total PDFGR-α expression. JHU-012, and −019 were grown alone or in 1:1 co-culture with MSCs for 6 days activation of PDGFR-α determined by Western immunoblotting. Total PDGFR-α was not detected by Western immunoblotting (*n* = 2).

Supplemental Figure 2 MSCs confer decreased cytotoxicity to cisplatin in OSCC. Representative photographs demonstrate crystal violet staining for JHU-012 **(A)** and JHU-019 **(B)** clonogenic assays as previously described and reported in Figure 4. Note the number of blue stained colonies within the wells. The black/violet staining clumps outside the wells are artifacts: crystallized crystal violet.

Supplemental Figure 3 MSC-induced cisplatin resistance is overcome by the PDGFR inhibitor, crenolanib in OSCC. Representative photographs demonstrate crystal violet staining for JHU-012 clonogenic assays following pretreatment with and without crenolanib as previously described and reported in Figure 5C.

Supplemental Figure 4 Total PDFGR-α expression. JHU-012 were grown alone or in 1:1 co-culture with MSCs were treated with either 0, 20, or 200 nM crenolanib for 6 days and activation of p-PDGFR-α determined by Western immunoblotting. Total PDGFR-α expression was not detected by Western immunoblotting at 0 and 20 nM crenolanib treatment (*n* = 2).
