# Supplementary material for: Activation PDGFR-α/AKT Mediated Signaling Pathways in Oral Squamous Cell Carcinoma by Mesenchymal Stem/Stromal Cells Promotes Anti-apoptosis and Decreased Sensitivity to Cisplatin
Source: Front Oncol. 2020 Apr 28;10:552. doi: 10.3389/fonc.2020.00552 (PMC7199219; doi:10.3389/fonc.2020.00552)

Supplemental Figures

Supplemental Figure 1.

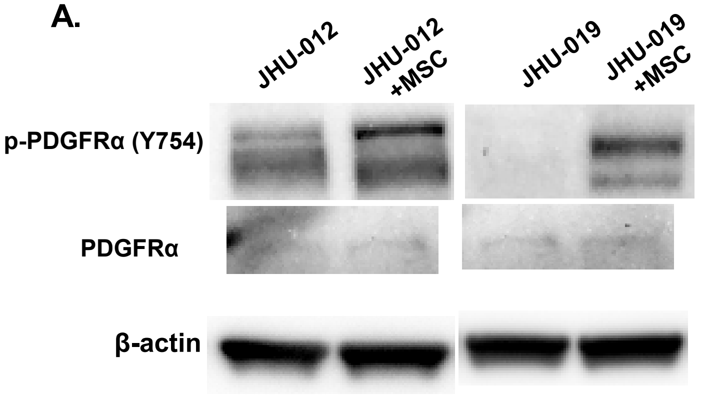

Supplemental Figure 2A

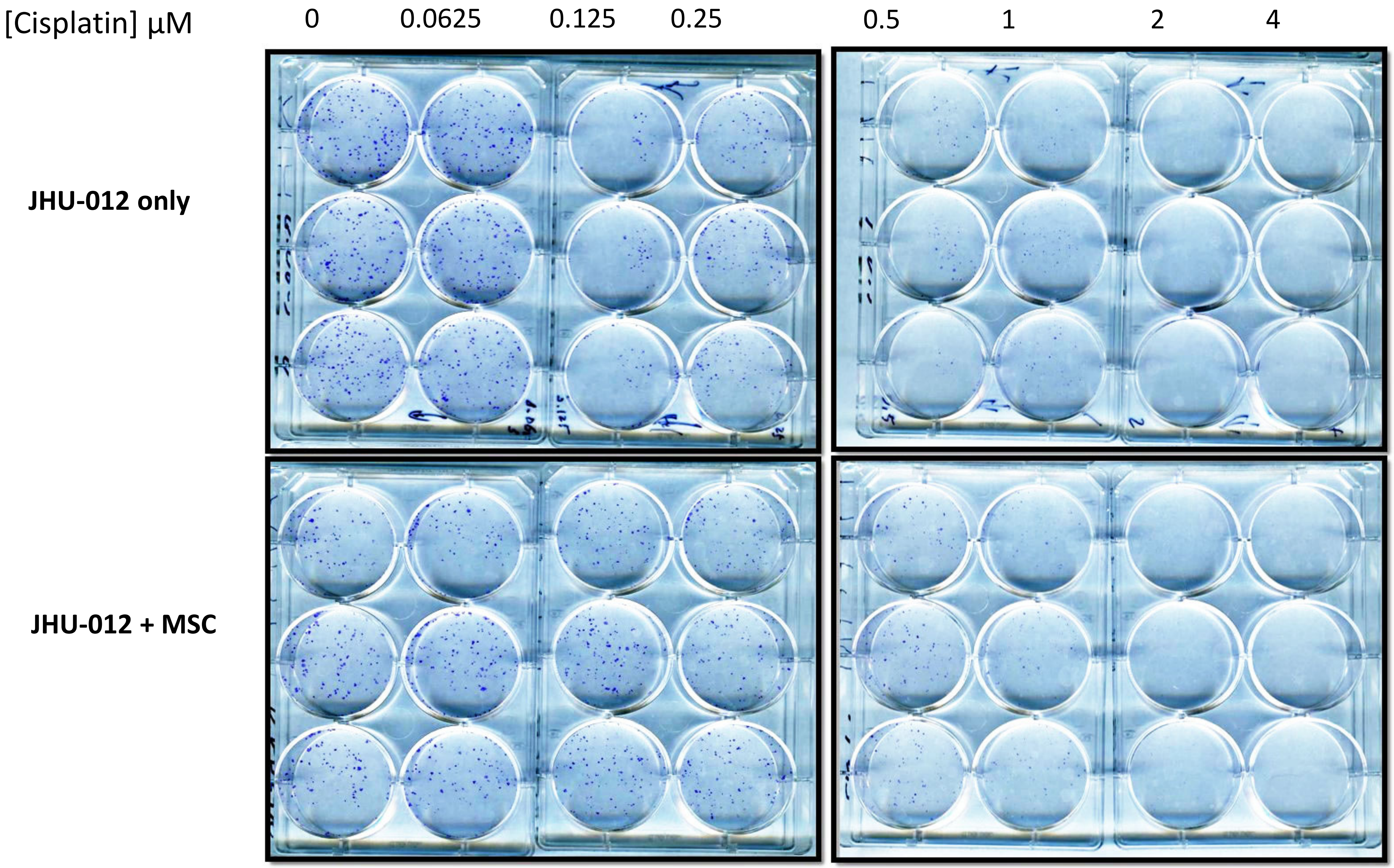

Supplemental Figure 2B.

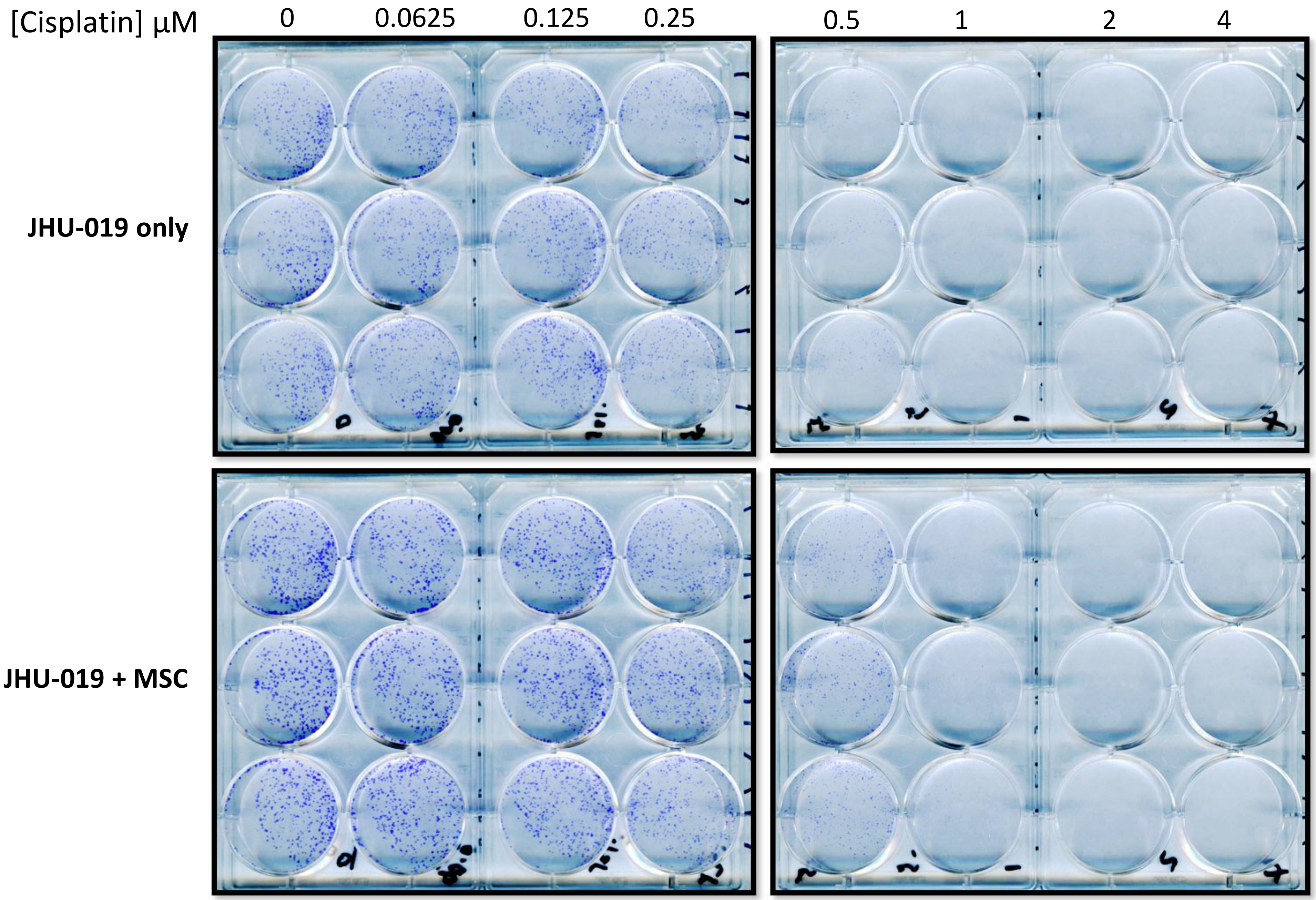

Supplemental Figure 3.

A.

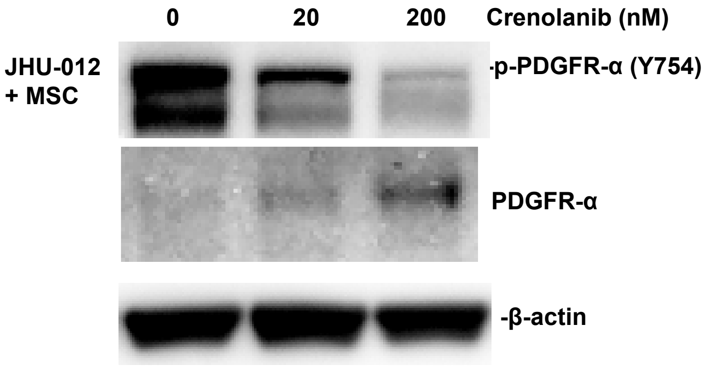

Supplemental Figure 4.

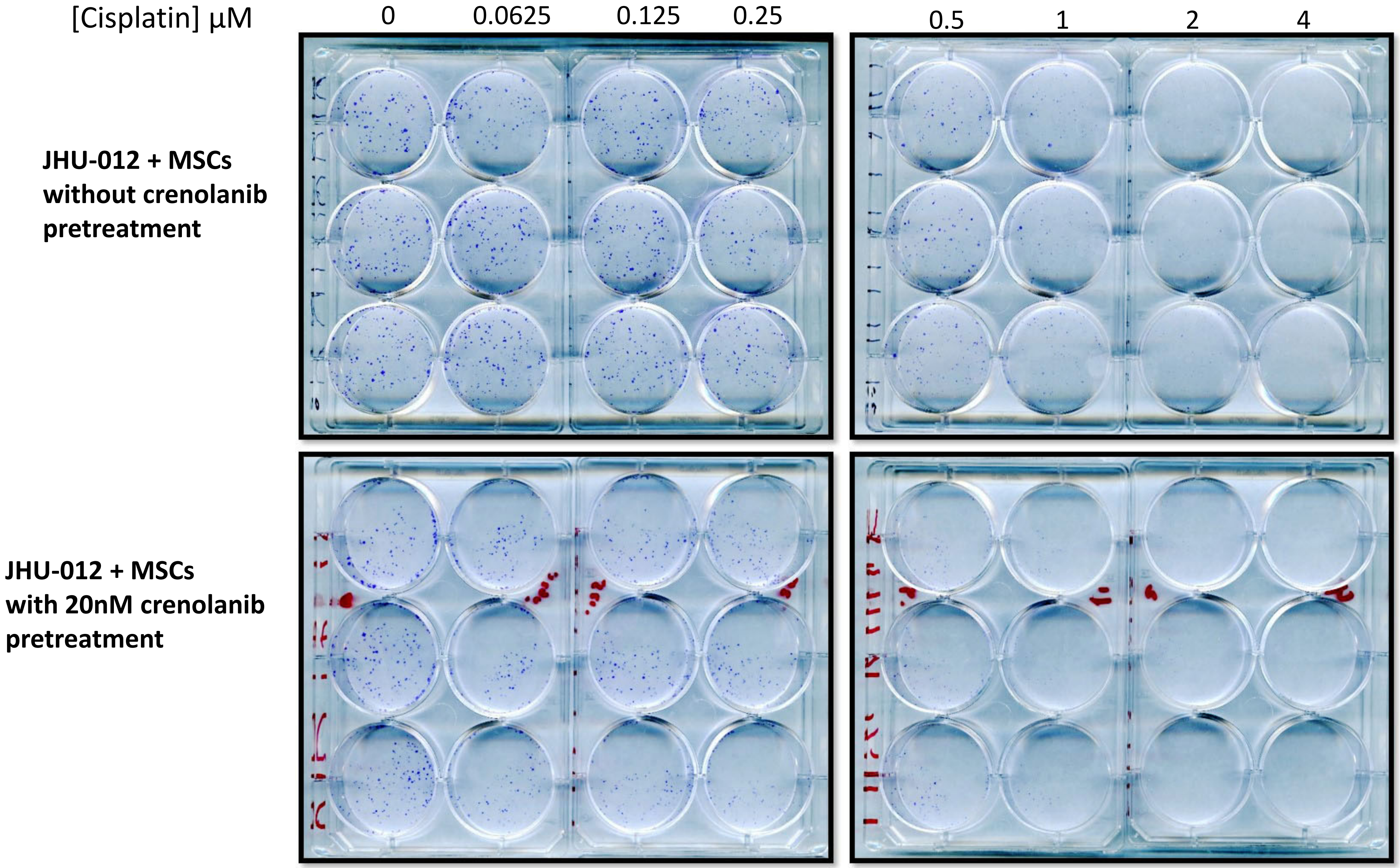

Supplement: Supplemental Figure 1 — Total PDFGR-α expression. JHU-012, and −019 were grown alone or in 1:1 co-culture with MSCs for 6 days activation of PDGFR-α determined by Western immunoblotting. Total PDGFR-α was not detected by Western immunoblotting (n = 2). [file Data_Sheet_1.PDF]
